# Changes in cigarette consumption and intention to quit in response to the COVID-19 pandemic in China

**DOI:** 10.18332/tid/160064

**Published:** 2023-03-09

**Authors:** Yimeng Mao, Yuchen Zhao, Michael Eriksen, Jidong Huang, Pamela Redmon, Claire Spears, Pinpin Zheng

**Affiliations:** 1 Department of Preventive Medicine and Health Education, School of Public Health, Institute of Health Communication, Key Lab of Public Health Safety of Ministry of Education, Fudan University, Shanghai, People’s Republic of China; 2 Department of Health Policy and Behavioral Sciences, School of Public Health, Georgia State University, Atlanta, United States; 3 Global Health Institute, Emory University, Atlanta, United States

**Keywords:** smoking, China, cigarette consumption, intention to quit, COVID-19

## Abstract

**INTRODUCTION:**

Significant changes occurred in the way people socialize and interact with each other since China reported its first COVID-19 cases. However, little is known about how smoking behaviors may have changed due to the COVID-19 pandemic in China. The aim of this study was to assess changes in smoking behavior and intention to quit during the early stage of the COVID-19 pandemic in China and to investigate the associated factors.

**METHODS:**

An online cross-sectional survey was conducted among Chinese adult smokers. Participants were recruited through snowball sampling from 19 March to 2 April 2020.

**RESULTS:**

A total of 1388 smokers participated in this study. Of those, 1014 (73.0%) reported not changing their cigarette consumption, 104 (7.5%) reported smoking more and 268 (19.3%) reported smoking less due to the pandemic. Average daily cigarette consumption among all participants decreased from 15.0 (IQR: 10.0–20.0) to 13.0 (IQR: 8.0–20.0) (W=6.919, p<0.001). For intention to quit, 270 (19.5%) respondents reported becoming more willing to quit, and 91 (6.6%) reported becoming less willing to quit. Multivariate analyses showed that tobacco addiction, overall knowledge about the relationship between smoking and COVID-19, level of attention devoted to COVID-19, anxiety, living alone, and number of smokers in the family were significantly correlated with cigarette consumption and intention to quit, and living alone was the strongest factor associated with increased cigarette consumption (AOR=5.29; 95% CI: 1.51–18.56).

**CONCLUSIONS:**

The COVID-19 pandemic led to a slight decrease in cigarette consumption and an increase in quitting intention among Chinese smokers. During the early stages of the pandemic, it was important to focus on the anxiety of smokers, dispel smokers’ misunderstandings of smoking and COVID-19 and create a supporting environment in the family to help smokers quit.

## INTRODUCTION

China was the first country to report cases of SARS-CoV-2 infection, which causes the coronavirus disease 2019 (COVID-19), in December 2019^1^. Because of the limited knowledge about SARS-CoV-2 and COVID-19 at the early stages of the COVID-19 pandemic and that no COVID-19 vaccines or treatments were available at the time, efforts to control the pandemic focused primarily on restricting the community spread of SARS-CoV-2 in early 2020. Many cities and provinces across China, for example, have implemented measures to restrict the community spread of SARS-CoV-2 since the beginning of 2020^2^. These measures included mandated quarantine for those with SARS-CoV-2 infection, stay-at-home orders, limiting travel except for essential activities, prohibiting crowd gathering, and mask mandates in public places^2,3^.

Smoking prevalence was high (50.5%) among Chinese men before the COVID-19 pandemic^4^. In addition, smoking plays an important role in socialization among some Chinese smokers. The COVID-19 pandemic and the resultant pandemic control measures reduced the opportunities for socialization, which may decrease smoking and cigarette consumption^5^. Furthermore, the knowledge and information about the relationship between smoking and COVID-19 may also influence smoking behaviors. For example, early reports showing smokers may be at higher risk of COVID-19 infection^6^, and many studies demonstrating that smokers were more likely to develop severe cases of COVID-19 than non-smokers, could lead to an increase in smokers’ motivation to quit smoking and a reduction in cigarette consumption among those who continue to smoke^7,8^.

However, the COVID-19 pandemic and the resultant pandemic control measures in China may also lead to changes that potentially increase smoking and cigarette consumption. A meta-analysis found that individuals experienced higher rates of depression (45%, 95% CI: 37–54) and anxiety (47%, 95% CI: 37–57) during the COVID-19 pandemic^9^. Smoking is often used as a coping mechanism for dealing with anxiety and depression; as such, smoking and cigarette consumption could potentially increase due to higher levels of anxiety and depression during the pandemic^10^. In addition, misinformation about the relationship between smoking and COVID-19, such as ‘smoking can prevent SARS-CoV-2 infection’ or ‘cigarette smoke can kill SARS-CoV-2’, may increase daily cigarette consumption among smokers who believe such misinformation^11,12^. Consequently, the overall impact of the COVID-19 pandemic and the resultant pandemic control measures on smoking and cigarette consumption may be mixed, and empirical evidence is needed to assess such an impact. Unfortunately, research that examines how the COVID-19 pandemic affects smoking in China is limited.

Identifying the overall influence of the early stage of the pandemic on smoking behaviors has both theoretical and practical significance for tobacco control in China. Previous studies using the data in the United States and Europe reported mixed effects of the pandemic on smoking behaviors in the early stage of the COVID-19 pandemic^13^. For example, a few studies reported increased interest in quitting and reduction in smoking among smokers in response to the pandemic^14^. However, other studies reported increased smoking rates and decreased quit attempts among smokers^15,16^. The number of studies examining the changes in smoking behaviors in response to the pandemic, specifically for Chinese smokers, is limited^17,18^. One study conducted in China in October 2020 found that more individuals chose to quit smoking and reduced the number of cigarettes they consumed daily after the outbreak of the pandemic^17^. By contrast, a second study conducted in May 2020 found no changes in cigarette consumption after the outbreak of COVID-19 in China. However, this study did not examine the factors associated with cigarette consumption^18^.

Our study aims to address this critical gap in the literature by assessing the changes in smoking behaviors and intention to quit among Chinese smokers early in the pandemic and identifying the factors associated with such changes. The findings from our study could provide insight into smoking-related behaviors during the pandemic in China and may help inform the design of educational campaigns and policies aimed at reducing tobacco use among Chinese smokers during the pandemic.

## METHODS

### Design and participants

This cross-sectional online survey was conducted using the Wenjuanxing platform (a popular online survey platform in China) from 19 March to 2 April 2020 (14 days). The inclusion criteria were: 1) aged >18 years; 2) smoked at least 100 cigarettes in their lifetime; and 3) smoked more than one cigarette in the month before the survey. Participants were recruited using snowball sampling via WeChat, the largest and most-used social media platform in China. The recruitment link was posted on WeChat with public access using the research team members’ ‘Friends Circle’, a function similar to posting public posts on Twitter and Facebook, which can be used to share personal photos or public website links in one’s ‘Moments (or Timeline)’ and make them visible to friends on the platform. The recruitment link could be further shared by anyone who saw the link in their respective ‘Friends Circle’, achieving additional snowball samples. Written consent was obtained from the eligible participants before they participated in the survey. The online survey included questions on demographics, cigarette consumption, intention to quit, quitting attempts, and two quality control questions. On average, it took participants approximately 5 minutes to complete the survey. In total, 1709 respondents from 31 provinces and autonomous regions in China participated in the online survey. After removing the invalid responses (e.g. excluding those who reported themselves as non-smokers in the survey and completed the survey in <2 minutes), 1388 (81.2%) participants were included in the current study.

### Measurements

*Cigarette consumption*

Changes in cigarette consumption were measured using the self-reported question: ‘Has your cigarette consumption changed since the COVID-19 outbreak?’. The response categories included: 1) smoking more, 2) smoking less, 3) having quit after the COVID-19 outbreak, 4) relapsed after the COVID-19 outbreak, and 5) no change. We categorized the answers into three categories: smoking more than before (including smoking more and relapsed), smoking less than before (including smoking less and quitting), and no change in smoking. Daily cigarette consumption before and after the outbreak of COVID-19 was measured by the following questions: ‘How many cigarettes did you smoke per day on average before the pandemic?’ and ‘How many cigarettes did you smoke per day on average after the pandemic?’. For those who reported decreasing or increasing cigarette consumption, two additional probing questions were asked about the reasons for decreasing/increasing their cigarette consumption after the pandemic.

*Intention to quit and quit attempts*

The following two questions measured the intention to quit. The first was: ‘Has your interest in quitting smoking changed recently because of the COVID-19 outbreak?’. Response categories included: 1) Yes, intention changed, 2) No change, still want to quit, and 3) No change, still not willing to quit. For the respondents who confirmed their change in quitting intention, a follow-up question was asked: ‘How has your intention to quit smoking changed as a result of the pandemic?’. Response categories were: 1) Did not want to quit smoking before, but now want to quit; 2) Wanted to quit smoking before, but no longer want to quit now; 3) Wanted to quit smoking before but the intention has decreased; and 4) Wanted to quit before and the intention has increased. Based on the responses to these two questions, the change in intention to quit was constructed with three categories as follows: 1) Intention to quit increased (did not want to quit smoking before, but now want to quit; wanted to quit before and the intention has increased); 2) Intention to quit decreased (wanted to quit smoking before, but no longer want to quit now; wanted to quit smoking before but the intention has decreased); and 3) Intention not changed.

Quitting attempts during the COVID-19 pandemic were assessed for those who made any quit attempts since the outbreak began, using the following two questions: 1) ‘How many times did you abstain from smoking for more than 24 hours during the pandemic?’, and 2) ‘What was the maximum duration (in days) of your abstinence since the outbreak began’.

*Covariates*

Knowledge about the relationship between smoking and COVID-19 was measured by four questions. The participants were asked about their opinions about the accuracy of four statements that were widely seen on Chinese social media in the early stage of COVID-19 in China. The response categories for each statement included ‘correct’, ‘incorrect’, and ‘not sure’^11,12^. These four statements were: 1) Smoking can prevent COVID-19; 2) Fine particles produced by smoking may increase the spread of the coronavirus; 3) Smoking could harm lungs and lead to severe consequences if infected by COVID-19; and 4) Cigarette smoking helps kill the coronavirus. Among these items, when participants chose ‘correct’ for statements 2 and 3, their responses were deemed correct. If participants chose ‘incorrect’ or ‘not sure’ for statements 1 and 4, their responses were deemed correct. A variable that captures the overall knowledge about the relationship between smoking and COVID-19 was constructed based on the responses to these four statements. This variable was coded into two categories: 1) at least one item was incorrect, and 2) all four items were correct.

Anxiety was assessed using the Chinese version of the Generalized Anxiety Disorder Scale (GAD-7), which assesses anxiety based on the Diagnostic and Statistical Manual of Mental Disorders (5th ed.)^19^. The reliability and validity of the Chinese version of the GAD-7 have been well-established by previous research^20^. There are seven items in the GAD-7, and the maximum score is 21. Anxiety was categorized as normal, mild anxiety, moderate anxiety and severe anxiety using the cutoff points of 5, 10, and 15, respectively^15^.

The following covariates were also included in this study: biological sex, age, education level (middle school and below, high school degree, associate degree, Bachelor’s degree and higher), and self-perceived severity of local COVID-19 epidemic. The last was assessed by the following question: ‘How do you think the severity of the COVID-19 epidemic in your city/locality compared with the overall situation in the country?’ with response categories ‘worse than average’, ‘similar to the average’, and ‘better than average’. Other covariates were the number of smokers in family (1 or >1), tobacco addiction (assessed using the heaviness of smoking index, HSI, with cutoff point of 4)^21^, amount of attention devoted to COVID-19 (measured by the question: ‘In the past month, to what extent did you pay attention to the COVID-19 related information?’ with response categories ‘high attention’, ‘moderate attention’, and ‘low attention’), perceived self-risk of COVID-19 infection (measured by the question: ‘How likely do you think you will become infected with COVID-19?’, with response categories ‘very likely’, ‘somewhat likely’, ‘not likely’), living with children (yes, no), and living alone (yes, no).

### Statistical analysis

Frequencies and proportions were computed for the changes in cigarette consumption and quitting intention, reasons for cigarette consumption changes, and the demographic characteristics. Median and interquartile ranges (IQR) were computed for daily cigarette consumption and the duration of abstinence. The kappa identity test was used to examine the consistency between daily cigarette consumption and self-reported changes in cigarette consumption. The related-samples Wilcoxon test was applied to examine the difference between participants’ daily cigarette consumption before and after COVID-19. Due to the failure to meet the requirements of the proportional odds assumption (p<0.001), the ordinary logistic regression model was not appropriate for the data. We used multinomial logistic regression to identify potential factors related to cigarette consumption and intention to quit. Adjusted odds ratios (AORs) and their 95% confidence intervals (CIs) were used to quantify the effects. Sex, age, education level, living with children, living alone, number of smokers in the family, tobacco addiction, overall knowledge, amount of attention devoted to COVID-19, perceived self-risk of COVID-19, anxiety, and local COVID-19 situation, were controlled for in all models. SPSS software version 20.0 (SPSS, Chicago, IL, USA) was used to conduct analyses. All tests were two-sided, and p<0.05 was considered statistically significant.

## RESULTS

### Demographic characteristics

Among the 1388 respondents, 1329 were males (95.7%), and 59 were females (4.3%), with an average age of 43.9 ± 12.0 years. One-third (33.2%) of the participants had a Bachelor’s degree and higher, and more than half of the participants (58.2%) lived in cities. Only 77 (5.5%) respondents felt that the COVID-19 situation in their local community was more severe than the overall situation in the country. Most participants (66.1%) had no other smokers living with them, and 85.1% of participants reported a low level of addiction to tobacco (Table 1).

**Table 1:** Descriptive statistics for the study sample of adult smokers in China, 2020 (N=1388)

*Characteristics*	*n*	*%*
**Sex**		
Male	1329	95.7
Female	59	4.3
**Education level**		
Middle school and lower	279	20.1
High school degree	273	19.7
Associate degree	375	27.0
Bachelor’s degree and higher	461	33.2
**Age** (years)		
<31	266	19.2
31–40	346	24.9
41–50	382	27.5
51–60	98	21.5
>60	96	6.9
**Severity of local COVID-19 epidemic**		
Worse than average	77	5.5
Similar to the average	297	21.4
Better than average	1014	73.1
**Numbers of smokers in family**		
1	917	66.1
>1	471	43.9
**Tobacco addiction**		
Low	1181	85.1
High	207	14.9
**Living with children**		
Yes	528	38.0
No	860	61.9
**Living alone**		
Yes	46	3.3
No	1342	96.6
**Amount of attention devoted to COVID-19**		
High	881	63.5
Moderate	428	30.8
Low	79	5.7
**Perceived self-risk of COVID-19 infection**		
High	367	26.4
Moderate	553	39.8
Low	468	33.7
**Anxiety**		
Normal	715	51.5
Mild anxiety	524	37.8
Moderate anxiety	103	7.4
Severe anxiety	46	3.3
**Overall knowledge**		
At least one incorrect	1136	81.8
All four items were correct	252	18.2
**Knowledge^a^**		
Smoking could harm lungs and lead to severe consequences if infected by COVID-19	916	66.0
Cigarette smoking helps kill the coronavirus	823	59.3
Smoking can prevent COVID-19	807	58.1
Fine particles produced by smoking may increase the spread of the coronavirus	450	32.4

a Participants answered correctly for each item.

### Cigarette consumption

Among the 1388 respondents in this study, 104 (7.5%) reported smoking more after the COVID-19 pandemic (including 30 who relapsed after the pandemic), and 268 (19.3%) reported smoking less than after the COVID-19 pandemic (including 72 who had quit due to the COVID-19 pandemic). Average daily cigarette consumption decreased from 15.0 sticks (IQR: 10.0–20.0) to 13.0 sticks (IQR: 8.0–20.0) during the early stage of the COVID-19 pandemic (W=6.919, p<0.001). The kappa identity test showed significant consistency between daily cigarette consumption and self-reported changes in cigarette consumption (p<0.001). Reasons for changes in cigarette consumption are listed in Table 2.

**Table 2:** Reasons for changing cigarette consumption among adult smokers in China, 2020

*Reasons*	*n*	*%*
**Reasons for decreasing cigarette consumption^a^**	268	100
My family didn’t allow me to smoke at home	207	77.2
I paid more attention to my health	186	69.4
My home is too small for smoking	182	67.9
Fewer opportunities to socialize with other smokers	171	63.8
It is inconvenient to smoke with a mask	170	63.4
It is inconvenient to go out to buy cigarettes	135	50.4
It is too much hassle to wash hands after going out to smoke	118	44.0
**Reasons for increasing cigarette consumption^b^**	104	100
My life was disrupted during the pandemic	59	56.7
I felt bored during the pandemic	58	55.8
I felt stressed during the pandemic	55	52.9
I had a bad mood during the pandemic	53	51.0
Life was impermanent and the most important thing was to enjoy life for the moment	30	28.8

a Only asked among smokers who reported smoking more than before.

b Only asked among smokers who reported smoking less than before.

### Intention to quit and quitting attempts

In all, 270 (19.5%) respondents reported becoming more willing to quit, and 91 (6.6%) respondents reported becoming less willing to quit, since the start of the COVID-19 pandemic in China. A total of 208 (15.0%) respondents reported they had tried to stop smoking for more than 24 hours during the COVID-19 pandemic. The maximum duration of abstinence among those who reported having made quit attempts ranged from 1 day to 60 days (median=5.0, IQR: 2.0–15.0).

### Factors associated with cigarette consumption and quitting intention

Table 3 presents the results based on the logistic regression. Factors associated with increased cigarette consumption (Model 1) since the start of the pandemic include: having mild (AOR=1.83; 95% CI: 1.14–2.95) and moderate/severe anxiety (AOR=3.90; 95% CI: 2.16–7.03); living in areas with similar (AOR=2.10; 95% CI: 1.32–3.34) or worse (AOR=2.40; 95% CI: 1.13–5.80) epidemic situations than the national average; and living with no children (AOR=2.18; 95% CI: 1.30–3.67).

**Table 3:** Factors associated with cigarette consumption and intention to quit (ITQ), results from logistic regression based on a sample of adult smokers in China, 2020 (N=1388)

*Variable*	*Model 1*	*Model 2*	*Model 3*	*Model 4*
	*Smoking more versus no change*	*Smoking less versus no change*	*ITQ increased versus no change*	*ITQ decreased versus no change*
	*AOR (95% CI)*	*AOR (95% CI)*	*AOR (95% CI)*	*AOR (95% CI)*
**Tobacco addiction** (Ref. Low)				
High	1.43 (0.85–2.41)	0.07 (0.03–0.19)**	0.42 (0.26–0.69)*	0.50 (0.24–1.03)
**Overall knowledge** (Ref. All four items were correct)				
At least one incorrect	1.05 (0.59–1.87)	0.49 (0.35–0.69)**	0.39 (0.28–0.54)**	1.68 (0.82–3.46)
**Amount of attention devoted to COVID-19** (Ref. Low)				
High	1.21 (0.48–3.00)	4.62 (1.63–13.10)*	2.48 (1.09–5.61)*	3.71 (0.88–15.76)
Moderate	1.08 (0.42–2.79)	3.44 (1.19–9.94)*	2.05 (0.89–4.73)	2.58 (0.59–11.38)
**Perceived self-risk of COVID-19 infection** (Ref. Low)				
High	1.49 (0.86–2.57)	1.59 (1.11–2.29)*	1.33 (0.93–1.91)	1.46 (0.82–2.61)
Moderate	1.03 (0.62–1.73)	0.99 (0.70–1.40)	0.90 (0.64–1.26)	1.27 (0.74–2.17)
**Anxiety** (Ref. Normal)				
Moderate anxiety or severe anxiety	3.90 (2.16–7.03)**	1.67 (1.03–2.72)*	1.82 (1.15–2.88)*	1.96 (1.00–3.84)
Mild anxiety	1.83 (1.14–2.95)*	1.59 (1.17–2.16)*	1.64 (1.20–2.22)*	1.45 (0.90–2.34)
**Severity of local COVID-19 epidemic** (Ref. Better than average)				
Worse than average	2.40 (1.13–5.08)*	1.35 (0.72–2.53)	0.79 (0.41–1.50)	1.13 (0.46–2.81)
Similar to the average	2.10 (1.32–3.34)*	1.35 (0.96–1.91)	0.89 (0.62–1.26)	0.80 (0.46–1.41)
**Education level** (Ref. Bachelor’s degree and higher)				
Middle school and lower	0.58 (0.30–1.15)	0.97 (0.63–1.50)	1.01 (0.66–1.55)	0.99 (0.52–1.89)
High school degree	0.76 (0.42–1.40)	0.84 (0.54–1.29)	0.74 (0.48–1.15)	0.98 (0.53–1.80)
Associate degree	0.92 (0.55–1.55)	1.04 (0.73–1.50)	1.29 (0.91–1.83)	0.77 (0.43–1.39)
**Sex** (Ref. Female)				
Male	0.98 (0.35–2.71)	0.51 (0.26–0.97)*	0.49 (0.26–0.92)*	0.86 (0.29–2.53)
**Age** (years) (Ref. >60)				
<31	0.84 (0.33–2.12)	0.95 (0.47–1.89)	1.57 (0.77–3.22)	1.94 (0.58–6.48)
31–40	1.23 (0.50–3.04)	0.95 (0.48–1.85)	1.45 (0.72–2.93)	1.87 (0.57–6.08)
41–50	0.60 (0.25–1.46)	0.84 (0.45–1.57)	1.36 (0.70–2.65)	1.80 (0.58–5.53)
51–60	0.45 (0.18–1.14)	0.93 (0.50–1.73)	1.08 (0.55–2.12)	2.29 (0.76–6.90)
**Living with children** (Ref. Yes)				
No	2.18 (1.30–3.67)*	1.04 (0.74–1.46)	1.04 (0.75–1.45)	1.09 (0.65–1.83)
**Living alone** (Ref. Yes)				
No	0.88 (0.36–2.13)	5.29 (1.51–18.56)*	2.82 (1.09–7.32)*	1.75 (0.39–7.80)
**Numbers of smokers in family** (Ref. 1)				
>1	1.01 (0.64–1.58)	0.56 (0.40–0.78)*	0.66 (0.47–0.91)*	1.06 (0.65–1.72)

AOR: adjusted odds ratio; adjusted for tobacco addiction, overall knowledge, amount of attention devoted to COVID-19, perceived self-risk of COVID-19, anxiety, local COVID-19 situation, education level, gender, age, living with children, living alone, numbers of smokers in family.

* p<0.05,

** p<0.001.

Factors associated with increased cigarette consumption (Model 2) include: being male (AOR=0.51; 95% CI: 0.26–0.97); having other smokers in the family (AOR=0.56; 95% CI: 0.40–0.78); having at least one incorrect belief about the relationship between smoking and COVID-19 (AOR=0.49; 95% CI: 0.35–0.69); and having high level of tobacco addiction (AOR=0.07; 95% CI: 0.03–0.19). Correspondingly, factors associated with decreased cigarette consumption (Model 2) include: paying moderate (AOR=3.44; 95% CI: 1.19–9.94) and high (AOR=4.62; 95% CI: 1.63–13.10) levels of attention to COVID-19; perceiving high level of risks of COVID-19 infection (AOR=1.59; 95% CI: 1.11–2.29); having mild (AOR=1.59; 95% CI: 1.17–2.16) and moderate/severe (AOR=1.67; 95% CI: 1.03–2.72); and not living alone (AOR=5.29; 95% CI: 1.51–18.56).

Factors associated with decreased intention to quit (Model 3) during the pandemic include: being male (AOR=0.49; 95% CI: 0.26–0.92); having at least one incorrect belief about the relationship between smoking and COVID-19 (AOR=0.39; 95% CI: 0.28–0.54), having a high level of addiction to smoking (AOR=0.42; 95% CI: 0.26–0.69); and having other smokers in the family (AOR=0.66; 95% CI: 0.47–0.91). Factors associated with increased intention to quit (Model 3) include: having mild (AOR=1.64; 95% CI: 1.20–2.22) and moderate/severe (AOR=1.82; 95% CI: 1.15–2.88) anxiety; devoting a high level of attention to COVID-19 (AOR=2.48; 95% CI: 1.09–5.61); and living with others (AOR=2.82; 95% CI: 1.09–7.32). No factors were found to be significantly associated with a decreased intention to quit (Model 4).

## DISCUSSION

This is one of the first studies to evaluate the change in cigarette consumption and quitting intention in the early stages of the COVID-19 pandemic across China. Our study found that, in general, Chinese smokers decreased smoking consumption and increased quitting intention during the early pandemic. The study also indicated that knowledge about smoking and COVID-19, attention to the pandemic, perceived self-risk, the local status of epidemic control and the number of smokers in the family were associated with such change. All of the above findings provide practical evidence for tobacco control-related education during the COVID-19 pandemic, which could be a ‘learnable window’ to encourage smokers to quit^22^.

Unlike the study of Liao et al.^18^ in May 2020, which found no changes in cigarette consumption after the outbreak of COVID-19 in China, the present study showed a significant decrease in daily cigarette consumption among smokers after the outbreak of COVID-19. The difference between the two studies maybe attributed to the gap in knowledge about the relationship between smoking and COVID-19. Our study proved that more knowledge about the relationship between smoking and COVID-19 was associated with the decrease in cigarette consumption. In the study of Liao et al.^18^, only 26.2% of current smokers agreed that smoking was a risk-factor for COVID-19 infection. However, this study indicated that 58.1% of participants disagreed that smoking can prevent COVID-19.

Family plays an important role in smoking behavior. This study found that living alone was a strong factor associated with more cigarette consumption and less quitting intention. This might be because smokers who live alone may smoke more due to boredom as there is lower communication with others. Also, they are less likely to receive complaints from family members during the stages of stay-at-home restrictions^11,23^. This is consistent with self-reports of participants that family members’ persuasion was an important reason for decreased cigarette consumption. However, the multivariate regression implied that when there were other smokers in family, participants were less likely to reduce cigarette consumption. Therefore, family members should encourage smokers to quit and create supportive environments to help them to stop smoking. Community health workers should pay more attention to smokers who live alone.

Our findings also indicate that more attention to the COVID-19 pandemic could help decrease cigarette consumption and enhance quitting intention, which is consistent with previous studies^14,24^. This is possible because high levels of COVID-19 attention could result in more related news seeking, from which smokers may improve their understanding of the risk of smoking and the importance of cessation during the pandemic^25-28^. Thus, media should take the role of informing the public about the risk of smoking during the pandemic.

However, some misunderstandings spread by the media about the relationship between smoking and COVID-19 may reduce one’s intention to quit, such as ‘Nicotine may be a treatment option for COVID-19’ and ‘Smoking can prevent COVID-19’^11,12,29,30^. These incorrect views mostly came from rumors spread by social media among smokers and have become part of smoking rationalization beliefs, which catered to smokers’ needs and helped them feel at ease while smoking^31-33^. As a result, smokers rationalize their smoking behaviors and are less likely to intend to quit^34^. It is the responsibility of the public health departments and the media to be good ‘gatekeepers’ and clarify the misunderstandings and rumors that have spread among smokers and have become smoking cessation barriers.

Among those related factors, anxiety was special because it could both enhance and decrease cigarette consumption. Previous studies indicated that anxiety caused by the changes in social contact, uncertainty about the future, job losses, and economic stress under the background of COVID-19 was positively associated with increased cigarette consumption^15,34,35^. Moreover, the present study also demonstrated that anxiety might decrease one’s cigarette consumption and motivate the intention to quit. We speculate that this anxiety was mainly caused by the focus on health. Such health-focused anxiety was common among current smokers because they had to face more severe consequences than non-smokers if infected with COVID-19^7^. Previous findings confirmed that health-focused anxiety might lead to increased quitting willingness^36^. Although anxiety may help to quit smoking, we discourage the media over emphasizing the consequence of COVID-19 infection among smokers, to avoid arousing severe anxiety. Besides, psychological institutions should also provide counselling services to help the public, including smokers, to develop a correct understanding of anxiety during the COVID-19 pandemic.

### Limitations

There are still a few limitations in this study. First, as a cross-sectional study, smoking intention and consumption before and during the COVID-19 pandemic were self-reported, resulting in recall bias. Second, the online survey may lead to selection bias, and the sample of this study was predominantly males with low tobacco addiction and better local COVID-19 control situations. Compared to more than 300 million smokers in China, the sample size of this study is really small. Thus, the sample may not be representative of the smoking population in China. Third, our study relied exclusively on self-reported data without biochemical verification. Further research may use longitudinal approaches and direct observation to explore additional evidence of the impact of the pandemic on smoking and other health-related behaviors.

## CONCLUSIONS

Our study found positive changes in cigarette consumption and quitting intention in response to the early stage of the COVID-19 pandemic in China. We believe that the reduction in cigarette consumption was associated with public attention to COVID-19 and perceived self-risk to infection. Knowledge about the relationship between smoking and COVID-19, as well as anxiety, may be related to intention to quit. It is essential for the media to inform the public about the risk of smoking and dispel related misunderstandings. Psychological institutions should provide counselling services to relieve the anxiety of smokers, and family members should encourage smokers to quit.

## Data Availability

The data that support the findings of this study are available from the School of Public Health, Fudan University, but restrictions apply on the availability of these data, which were used under license for the current study, and so are not publicly available. The data are, however, available from the authors upon reasonable request, with permission of the School of Public Health, Fudan University.
